# Implications of Convolutional Neural Network for Brain MRI Image Classification to Identify Alzheimer's Disease

**DOI:** 10.1155/2024/6111483

**Published:** 2024-08-22

**Authors:** Ananya Yakkundi, Radha Gupta, Kokila Ramesh, Amit Verma, Umair Khan, Mushtaq Ahmad Ansari

**Affiliations:** ^1^ Department of Computer Science and Engineering Dayananda Sagar College of Engineering, Bangalore, Karnataka, India; ^2^ Department of Mathematics Dayananda Sagar College of Engineering, Bangalore, Karnataka, India; ^3^ Department of Mathematics Faculty of Engineering and Technology Jain (Deemed-to-be University), Bangalore, Karnataka, India; ^4^ Department of Computer Science & Engineering University Centre for Research & Development Chandigarh University, Gharuan, Mohali 140413, Punjab, India; ^5^ Department of Computer Science and Mathematics Lebanese American University, Byblos, Lebanon; ^6^ Department of Mathematics Faculty of Science Sakarya University, Serdivan, Sakarya 54050, Türkiye; ^7^ Department of Mechanics and Mathematics Western Caspian University, Baku 1001, Azerbaijan; ^8^ Department of Pharmacology and Toxicology College of Pharmacy King Saud University, Riyadh 11451, Saudi Arabia

## Abstract

Alzheimer's disease is a chronic clinical condition that is predominantly seen in age groups above 60 years. The early detection of the disease through image classification aids in effective diagnosis and suitable treatment. The magnetic resonance imaging (MRI) data on Alzheimer's disease have been collected from Kaggle which is a freely available data source. These datasets are divided into training and validation sets. The present study focuses on training MRI datasets using TinyNet architecture that suits small-scale image classification problems by overcoming the disadvantages of large convolutional neural networks. The architecture is designed such that convergence time is reduced and overall generalization is improved. Though the number of parameters used in this architecture is lesser than the existing networks, still this network can provide better results. Training MRI datasets achieved an accuracy of 98% with the method used with a 2% error rate and 80% for the validation MRI datasets with a 20% error rate. Furthermore, to validate the model-supporting data collected from Kaggle and other open-source platforms, a comparative analysis is performed to substantiate TinyNet's applicability and is projected in the discussion section. Transfer learning techniques are employed to infer the differences and to improve the model's efficiency. Furthermore, experiments are included for fine-tuning attempts at the TinyNet architecture to assess how the nuances in convolutional neural networks have an impact on its performance.

## 1. Introduction

The chronic clinical condition called Alzheimer's disease (AD) is a neurological disease, which is diagnosed through assessments such as MRI and positron emission tomography (PET). These assessments are the studies of specific patterns in the brain of patients with the Alzheimer disease which shows characteristic changes in the initial, medium, and advanced stages of the disease. In 1907, Alois Alzheimer described about AD and it was a rare disease during that period. In the present scenario, AD has become a common disease for the age group above 60 years.

MRI has a predominant role to play in AD due to its characteristics which helps patients to get their diagnosis with minimal discomfort [1]. Using MRI to accurately classify clinical findings in AD is a crucial and essential stage in the treatment process. However, because of the similarities found in brain pictures, this classification is prone to numerous misclassifications [2]. The early phases of AD have a slow onset, making the diagnosis difficult. However, when the illness worsens, it significantly impairs day-to-day functioning and results in irreparable brain damage. This places a heavy load on healthcare systems as well as the relatives of patients [3]. With the existing drugs merely able to halt the disease's progression, there are currently no effective clinical techniques for the prevention or treatment of AD. As a result, one of the most important issues facing the medical community and society at large is the early detection of AD [4, 5].

In the field of image classification, a lot of work has taken place using convolutional neural networks (CNNs). The CNN has found applications in the field of computer vision since its inception in the 1990s in a wide variety of fields that include image classification, object detection, and action recognition. A decline in their usage was observed when support vector machines rose to power with higher accuracy and comparatively easier implementation. In 2012, the authors in [6] brought prominence back to CNNs by showing substantially higher image classification accuracy on the ImageNet large-scale visual recognition challenge (ILSVRC) [7] using a deep, large neural network. Since then, [6]'s deep model has been used as a backbone in several high-end problems and has proven to be a magnificent innovation. The availability of large-scale data and the applicability of deep convolutional networks have made computer vision a rapidly evolving field motivating revolution henceforth. Visual recognition, object detection, spatial and temporal pyramids, and feature pyramids [8–12] are some of the many revolutionary innovations that have evolved through the recent years paving way for similar applications to rise and grow.

Convolutional neural networks, predominantly used for feature extraction from visual data, convert them into feature maps which can be used in the subsequent layers to make predictions. Unlike deep neural networks (DNNs), CNNs do not require human supervision as they are capable of identifying the important features from the given data. In addition, CNNs are size-oriented performers—CNNs with larger receptive fields locate objects of larger scale and the ones with smaller receptive fields are focused on smaller objects. CNNs, not limited to images, can be applied to videos and text as well, by adjusting the dimensions and parameters as per the requirements [13]. Among others, one drawback of CNNs is that they are computationally expensive when applied to large-scale data of high-resolution images. With this in view, a literature survey was conducted on the application of CNNs on AD which is discussed in the next section.

## 2. Literature Survey

According to the latest WHO data published recently in 2020, Alzheimer's disease and dementia cause nearly 1,00,000 deaths in India, comprising close to 1.5% of the deaths in the nation. Signs of the trend declining are scarce. Identifying Alzheimer's disease from brain MRI images requires professional MRI technologists which is rare and draws huge expenses, especially in the rural regions of the country. Fraudulent cases are ample in both rural and urban areas. By the year 2050, it is predicted that one out of three senior citizens succumb to Alzheimer's disease or any other kinds of dementia. To battle this quandary, a lot of work was carried out that classified brain MRI images into infected or not, and some of those are discussed as follows:

The authors in [6] proposed a large, deep convolutional network for image classification on the 1000 category dataset of ImageNet [14], in which they were able to achieve error rates (complement of the accuracy rate) as low as 37.5% and 17%. The network is made up of 60 million parameters and 650,000 neurons, consisting of five convolutional layers, followed by three fully connected layers with a final 1000-way softmax layer. A dropout layer has also been employed to avoid overfitting. The fully connected layers tailing CNNs are made up of 4096 neurons each. A stride value of 4 is used to help with the generalization of the image input. This architecture has since been used as a backbone in various applications that include object detection and image segmentation.

SPP (Spatial Pyramid Pooling)-nets [15] have shown greater accuracy in image classification and object detection as well, by solving a problem that arises when dealing with convolutional neural networks. SPP-net uses the same architecture as that of [6] (among various others), but the difference is that of the use of pyramid pooling layers. CNNs convert input images into feature maps of different scales and sizes, complicating the process for fully connected layers. Pooling layers convert the feature maps into a fixed-length feature vector as input for fully connected layers that follow to do the predictions. Training the images on the dataset of ILSVRC 2014, they rank third in performance with an error rate of 8.06%. During training, the images are cropped into 224 × 224, followed by horizontal flipping and colour altering as a data augmentation strategy to enhance semantic understanding of the image data. Max pooling layers are used after the second and third convolutional layers of the architecture. It is further elucidated how multilevel and multisize pooling improve accuracy and full-image representation of images has a positive impact on accuracy.

The idea in [16] is to use large, deep convolutional networks for mainly object detection, with image classification being a subset of it. It is a two-step object detection method where in the first step, regions are proposed and are then classified to further carry out object detection. To address the conflict of translation invariance for image classification, they propose position-sensitive score maps. This method achieved 83.6% mean average precision on the PASCAL VOC dataset for object detection. Since deeper levels in a large network are less sensitive to translation, RoI layers are added, post which, the layers are no longer translation invariant. To subsume translation variance into the FCN, they have constructed a set of position-sensitive score maps by using a bank of specialized convolutional layers as the FCN output. Each of these score maps encodes the position information concerning a relative spatial position. The R-FCNs are trained to classify the RoIs into object categories and backgrounds. All learnable weight layers are convolutional and are computed on the same image. The last convolutional layer learns the position-sensitive score maps.

Being coherent and robust [17] to almost every large dataset such as the one in [18] and ILSVRC [19], the primary shortcoming of these ConvNet architectures [6, 15, 16] is that they take a lot of time for training. Their speed at run-time is impeccable with only tens of frames per second. However, the training procedure takes days on the best of GPUs worldwide because of the profundity of the data. Feasibility is at risk since GPUs are expensive. The weights of several layers of ConvNets are to be adjusted to get at least credible outcomes. With the availability of datasets for medical purposes being meagre, training in such humongous architecture would not be adept. Most likely, the model may overfit on the dataset, going through the same inputs frequently and tweaking the weights of the neuron connections irregularly. In other words, CNNs tend to memorize the data rather than learn it when trained over the same data incessantly. In contrast, the lack of large-scale data may even lead to underfitting, which means that it may not understand the data wholly and would fail to make the right predictions.

In a fairly recent study, deep learning techniques were applied to a brain MRI dataset in [20]. With this method, the authors identified the different types of diseases, including AD, by performing multilevel classification utilizing transfer learning in conjunction with VGG-16 and Fastai. Nevertheless, there is an overfitting issue with this method. Similarly, the authors in [13] suggested an SVM-based model for using structural brain MRI to classify AD. To make precise AD predictions in this work, MRI data were integrated with the SVM model. Nonetheless, a common shortcoming of SVM is its inability to select the proper kernel function.

In a similar vein, the effectiveness of the Pareto-optimized VGG model was examined in contrast to traditional VGG variants; this study was carried out to evaluate the deep learning model's potential for extracting important characteristics from MRI and PET data, as evidenced by their capacity to extract critical features [21]. Nevertheless, patients with mildly damaged functional brain networks cannot have changes in their brain networks identified by this approach [22]. To classify AD at different phases, the authors in [23] suggested an accurate technique based on transfer learning. This method divides the brain into four categories: normal, early-mild, late-mild, and AD. To do this, they used the standard MRI dataset of AD and segmented the tissues to extract the grey matter. We tuned the architecture using the grey matter while freezing the different types of characteristics. If the classification layer is unable to discern between the many categories for a given issue, this strategy might not work as intended [24].

Spectral GPT [25], a technique introduced in early 2024, is a foundation model that revolutionizes visual representation learning, through the enhancement of generative pretrained transformers to clean the patterns in visual data. Similarly, many techniques that rely on transformers for image classification have been put forth, making the field of image classification a growing necessity for research. However, these techniques use large amounts of data and powerful GPUs to perform computations, which is not feasible for everyone.

It is safe to conclude that not all large architectures are suited for every kind of classification problem. Sometimes the smaller models perform well and defeat the purpose of using a larger one. Hence, small models that serve a specific purpose of image classification for small datasets are found to be powerful and efficient. This paper corroborates how a tiny, simple network can have higher accuracy with the right parameters than a large, deep network [26] with millions of parameters when trained on a small amount of data. In simple terms, it is elucidated how the fine-tuning of the convolutional network can impact its usability. When hardware accelerators and substantial amounts of data are unavailable, TinyNet can provide state-of-the-art accuracy for image classification. The model developed in regard to this paper will be compared with some of the standard models such as ResNet and EfficientNet to further substantiate the argument.

## 3. Dataset and Data Collection

**Figure 1:**
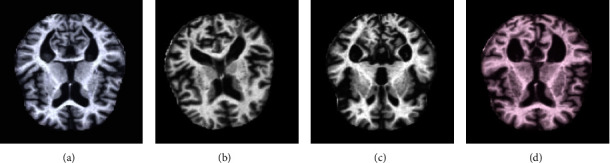
The dataset consists of four categories of images: (a) nondemented; (b) mild demented; (c) moderate demented; (d) very mild demented.

The “augmented Alzheimer MRI dataset” has been collected from Kaggle (open source) [27]. The dataset consists of two directories of MRI-scanned images, namely, the original dataset and augmented Alzheimer dataset. Each of these directories is divided into four more directories of images, each belonging to one class, namely, “nondemented,” “mild demented,” “moderate demented,” and “very mild demented.” The additional undertaking of bifurcating the images into classes before training is avoided since the dataset is already divided into directories of classes. An example image of each of the different classes is shown in Figure 1 with their respective labels.

With a total of 40,000 images, the augmented Alzheimer dataset directory consists of roughly 34,000 images and the original dataset directory consists of 6,000 images. The augmentation used to obtain the augmented Alzheimer's dataset is quite simple and will be spelled out in further sections.

**Figure 2:**
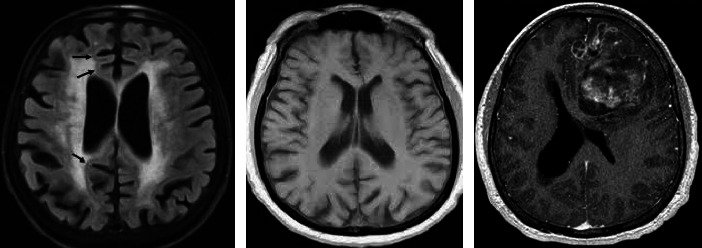
The above images are a few of the custom images extracted from Google to validate the model. Our model was able to classify the images into the correct categories during test time.

Since all the images above look wildly similar to the naked eye, the need for their classification using machines has become a necessity. Each directory in the augmented Alzheimer dataset contains about 8000 images for training purposes. To avoid the biases in collecting the images from one source, a few custom images are extracted from Google to validate the model which is explained in the next section as shown in Figure 2.

## 4. Methodology

**Figure 3:**
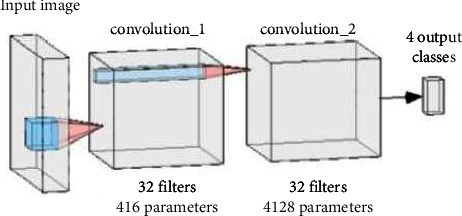
Block diagram representation of the network with its layers and the parameters with 4 output classes: nondemented, mild demented, moderate demented, and very mild demented.

The neural network presented in the paper consists of two 2D convolutional layers for feature extraction followed by a fully connected layer with sigmoid activation. A flattened layer is used after the convolutional layers to resize the feature maps to match the size of the fully connected layers. Pooling layers were omitted because they introduced information loss. Each convolutional layer consists of 32 filters with kernels of size 2. The use of a larger kernel size put forth a decline in accuracy and also increased computational complexity by taking too much time and memory. In the case of a smaller kernel size, weights are shared among the multiple layers of the network while reducing the computational costs. The benefit of feature sharing is that overfitting is reduced and semantic understanding is improved. For each layer of convolution, the ReLU activation function was utilized, owing to its faster convergence and higher generalization capability. The ReLU activation layer also prevents exponential growth, nevertheless speeding up training. Sigmoid activation for convolutional layers promotes overfitting and increases convergence time. The block diagram of the network used in this paper for the present study is shown in Figure 3.

In this paper, a comparative study of the performance of the different pretrained networks on the MRI dataset to classify the images of the four categories mentioned in the data section has been carried out. These pretrained networks are evaluated for their efficiency during the classification, and the results are shown in Table 1.

Measures that are both objective and subjective are used to assess the dependability and efficiency of models and approaches used in feature extraction and classification. Researchers mostly employ objective measurements to effectively compare the strategies. A few often-used metrics for image categorization are weighted average, recall, accuracy, precision (pre), and F1-score. In the event of supervised learning, these metrics are dependent upon statistical measurements (such as true positive (TP), true negative (TN), false positive (FP), and false negative (FN)) derived from the confusion matrix, contingency table, or error matrix. In this study, we have used most efficient measures such as accuracy and the Matthew's correlation coefficient (MCC) which is a more dependable statistical rate that provides an excellent result.

**Figure 4:**
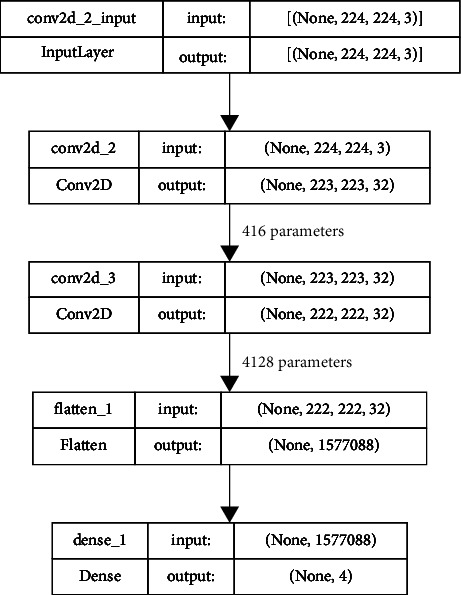
Details of the layers used in the network with the number of parameters distributed in each layer.

The number of parameters used in this model in different layers is represented in Figure 4. This figure provides the complete details of the layers and the parameters used in each layer with proper demarcation. As mentioned in Figure 3, there are 2 convolutions having 32 filters each with the input and the output layers.

**Figure 5:**
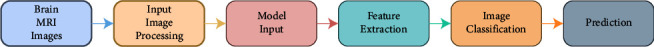
Proposed schematic diagram for Alzheimer's detection from brain MRI signal.

As observed in Table 1, the accuracy of the model and the MCC score are better compared to any other standard network models used for image classification. The block diagram of the process involved in classifying the MRI images is shown in Figure 5.

The stride and padding, among several other parameters of 2D convolutional layers, are set to the default values of 1 and valid, respectively. A subtle increase in the stride and padding values showed a negative effect on the accuracy of the experiments and with such unbefitting parameters, generalization would take forever to converge. The tensor flow flattened layer is used to reduce the multidimensional output of convolutional layers to a single dimension for the fully connected layers. For the only fully connected layer in the network, an activation function of “sigmoid” was used. The use of “softmax” for activation showed a drop of 5 percent in accuracy, which for datasets of such scale is significant. Other activations do not serve the purpose of classification well and are thus not ventured into the network.

### 4.1. Loss

The loss function “categorical cross entropy” is best suited for multiclass classification. Despite its common employment with “softmax” output, this loss function proves to be very efficient with our model with “sigmoid” output. In several other references [6, 16, 26, 28], the activation of “softmax” has been used because of its greater generalization abilities.

### 4.2. Optimizer

An implementation of the Adam algorithm is used for optimization. The algorithm is a stochastic gradient descent method based on adaptive estimation of first-order and second-order moments mentioned in [27]. The method is computationally efficient, has little memory requirement, and is invariant to diagonal rescaling of gradients. Though it is more suited for larger networks, it has proved to be a great aid in small networks, too. The learning rate was set to a default value of 0.001 since small changes caused severe fluctuation in the accuracy rate.

**Figure 6:**
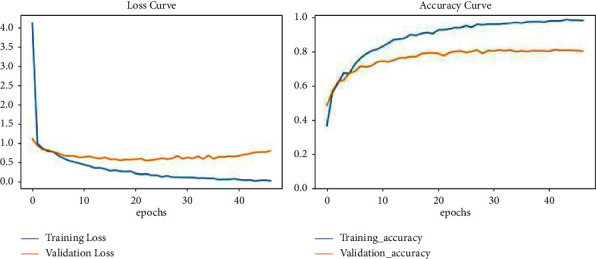
Loss and accuracy curves depicting error variance and the accuracy of the model during training and validation MRI datasets in blue and red colour curves, respectively, for 50 epochs.

Finally, the model was evaluated on the “accuracy” metric. Accuracy and loss curves are plotted using Python libraries for visualization.  Figure 6 visualizes error variance and the accuracy of the model during training and validation of the images represented in blue- and red-colour curves, respectively, for a number of 50 epochs.

## 5. Results and Discussion

**Figure 7:**
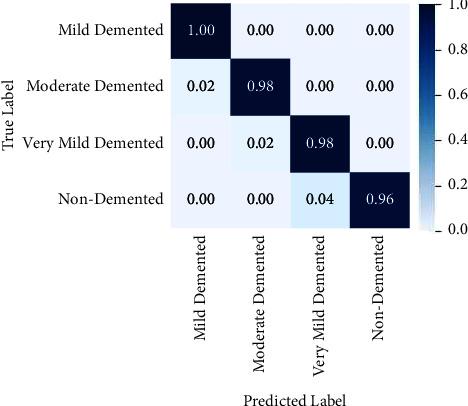
Confusion matrix calculated between the true and the predicted labels for nondemented, mild demented, moderate demented, and very mild demented classes.

Alzheimer's disease has become prevalent in the present world. To address this issue, augmented Alzheimer MRI dataset has been collected and segregated in which training and testing datasets are divided in the ratio of 80 : 20. This is a standard segregation of the datasets that has been implemented in deep learning tasks, ranging from a simple image classification to a most complex task such as object detection or semantic segmentation. The major benefit of this step is that it helps to prevent overfitting by improving generalization and provides a validation dataset to vouch for the model. Keeping in mind that determining the optimal number of epochs is an important part of the process, training is performed on the dataset consisting of 28K images on TinyNet. Training for a low number of epochs might hinder the model from achieving its best performance, whereas too many epochs may lead to accuracy plateauing after a certain threshold value. The right number of epochs is determined by using a tensor flow callback method called early stopping, which stops training the model when the accuracy does not improve for a certain number of epochs. This way we can find the minimal number of epochs needed to achieve the best accuracy for a model. Using this technique, an accuracy value of 98% was achieved by training the model for 50 epochs with an error rate of 2%. The proposed method uses a combination of forward and backward recursion techniques to choose only a subset of features. The partial *Z*-test values are used in the forward recursion approach to identify the most connected features, and in the backward recursion method, the least correlated features are eliminated from the same feature space. The *Z*-test values are evaluated in each scenario utilizing the given disease labels and are shown in the confusion matrix given in Figure 7.

**Figure 8:**
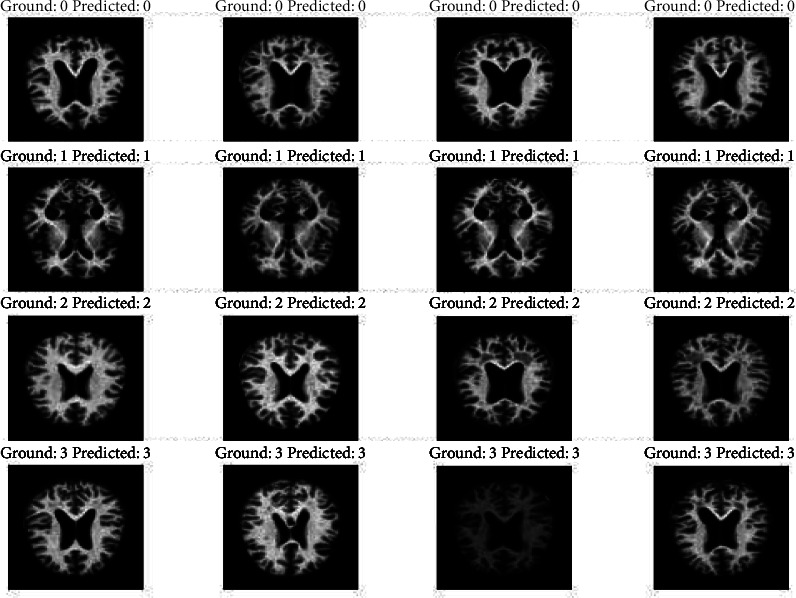
The predicted results against the ground truth for the test dataset trained using the model developed in the study.

**Figure 9:**
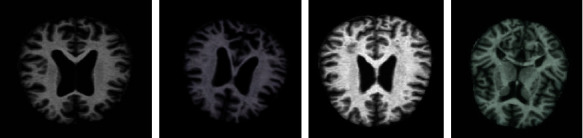
The images shown here are collected from the testing directory for validation purposes.

The capacity of the suggested method to quickly and accurately identify localized features is one of its strongest points. The outcomes of the predictions compared to the actual data are displayed in Figure 8. For validating the model, the remaining dataset of 6K images is used. The original dataset would be used for testing purposes only and thus is not split any further. The model validation is computed parallelly using the validation dataset. The number of samples to be taken per epoch is also predetermined to avoid overloading the model. The model attained a validation accuracy of 80% with an error percentage of 20%. For the validation of the model, the images captured during the testing of the model are shown in Figure 9.

Custom data from Kaggle were retrieved to test the model's performance. As an extra step of evaluation, a COVID-19 dataset of chest scans was also collected and tested. The model obtained 80% accuracy on the test set. To compare the CNN model performance with other existing models, a comparison of the proposed TinyNet architecture model for Alzheimer's disease identification using the MRI dataset is shown in Table 2.

The method used in the present study uses a smaller number of layers and parameters showing better efficiency in training the datasets collected from Kaggle in comparison with the existing models referred to in Table 2. This ensures that the results obtained are not spurious. Furthermore, the evaluation of the model used in this study applied to the bird classes' dataset leads to overfitting. The training accuracy reached well over 85% accuracy, while the validation plummeted at 30% accuracy. This accounts for the argument that TinyNet works well on smaller datasets with a lesser number of classes.

The main challenge with the training of this model was the lack of data. Though there are plenty of resources available for collecting the data, the main aim of this paper was to develop a model that does not rely on the intricacies of the data. In doing so, the model may fail to identify the minute details that indicate Alzheimer's disease in a person. Thus, the model might fail to recognize the early stages of dementia, thereby reducing its reliability.

## 6. Conclusion

TinyNet, as the name suggests, is a small convolutional model that can be used to train small datasets, the main difference from the existing technologies being its small size and commendable performance that can be extracted without the usage of powerful GPUs. This network is tested for only small-size images due to its structure and for the dataset used in the present study, specifically in pretrained networks, it performs better for the same learning rate 0.001 and 50 epochs. Due to the increase in the number of Alzheimer's patients as per WHO surveys, our experiments are primarily focused on the classification of brain MRI images to detect Alzheimer's disease in patients. The work has been concentrated on identifying the initial stages of dementia so that it can be treated before it spreads. It is substantiated that large models do not always provide the best results when used for small datasets like the one used in this experiment. A publicly accessible standard brain MRI dataset was used to test and validate the suggested method, and the results indicated a weighted average identification rate of 98% with an MCC score being 0.98 when compared to the most advanced systems. Certain technologies may become essential tools in the healthcare industry so that doctors may accurately diagnose Alzheimer's disease. Since the suggested method is tested and proven on a limited sample size, we will also evaluate the suggested system's overall performance in the future by examining a sizable sample size.

## Figures and Tables

**Table 1:** Performance of pretrained networks on MRI datasets.

Sl no	Network	Optimizer	Epochs	Learning rate	Accuracy (%)	MCC score
1	TinyNet	Adam optimizer	50	0.001	98	0.98
2	ResNet	Adam optimizer	50	0.001	89.27	0.94
3	EfficientNet	Adam optimizer	50	0.001	94.52	0.97
4	GoogleNet	Adam optimizer	50	0.001	91.13	0.95
5	InceptionNet	Adam optimizer	50	0.001	95.33	0.97

**Table 2:** Comparison of the approach used in the present study with the existing models.

Research literature	Methodology	Accuracy rate (%)	MCC	Error rate (%)
[29]	Dementia network and CNN	89.5	0.94	10.5
[30]	CNN using transfer learning	93.4	0.95	6.6
[31]	ResNet50 with multiphantom convolution	90.5	0.94	9.5
[22]	Transfer learning	92.4	0.95	7.6
[32]	CNN	88.1	0.93	11.9
[33]	ResNet, VGG, and transfer learning	80.9	0.89	19.1
[34]	CNN	77.8	0.85	22.2
[35]	CNN, VGG-16, and VGG-19	82.5	0.88	17.5
Proposed model	TinyNet and CNN	98	0.98	2

## Data Availability

The datasets used and/or analyzed during the current study are available from the corresponding author upon reasonable request. In addition, no human data are used in this manuscript.
